# Acute Orbital Symptoms Revealing Metastatic Neuroendocrine Neoplasm: A Case Report

**DOI:** 10.51894/001c.165615

**Published:** 2026-07-31

**Authors:** Amina Ibrahim, Alyssa Gappy, Seynab Farah, Jasmine Philipose, Angela Peterson, Myles Jerrett

**Affiliations:** 1 Henry Ford - Wyandotte

## 87

### INTRODUCTION

Metastatic Grade 2 neuroendocrine neoplasms (NENs) involving the orbit are rare and may present with diplopia, proptosis, and restricted extraocular movements. Although uncommonly diagnosed in the emergency department (ED), these presentations warrant orbital CT imaging. This case highlights the importance of a thorough history and physical examination in ED patients with ocular complaints to determine whether orbital CT is indicated. In the ED, CT is most commonly used to evaluate acute trauma, suspected orbital cellulitis or abscess, and acute vision loss.

### CASE DESCRIPTION

A 58-year-old female with no significant past medical history presented with acute right eye pain after sleeping in her contact lenses. She reported right eye swelling and a mild right-sided headache. Examination revealed a markedly injected right eye with significant chemosis, preserved visual acuity, and intact extraocular movements. CT of the orbits demonstrated orbital cellulitis, a prominent right superior ophthalmic vein, and a 4.2-cm soft tissue mass involving the superior rectus muscle. Due to concern for an infiltrative orbital process and potential progression to orbital compartment syndrome, the patient was transferred for ophthalmologic evaluation. She subsequently developed 360-degree chemosis, restricted extraocular movements, and findings concerning for orbital apex syndrome, including an afferent pupillary defect, ophthalmoplegia, and decreased sensation in the V1 distribution. She was given intravenous methylprednisolone, vancomycin, and ampicillin–sulbactam, with clinical improvement. CT of the chest, abdomen, and pelvis revealed multiple axillary, subpectoral, and mediastinal lymph nodes. Lymph node biopsy confirmed metastatic neuroendocrine tumor. The patient was discharged on prednisone with ophthalmology and oncology follow-up. Oncology initiated treatment through the NETTER-3 trial for first-line therapy of metastatic Grade 2 NEN.

### DISCUSSION

This case highlights the importance of maintaining a high index of suspicion when orbital symptoms deviate from typical presentations. The patient’s initial history suggested a common diagnosis such as corneal abrasion or keratitis. While many ocular complaints in the ED do not require imaging, concerning findings such as significant chemosis, eyelid swelling, and pain should prompt CT imaging. In this case, imaging was critical in identifying an underlying neoplastic process, emphasizing the importance of a thorough eye exam in patients with atypical orbital findings.

